# Osteofibrous Dysplasia Involving Both Tibia and Fibula: A Rare Campanacci‑Type Presentation

**DOI:** 10.5334/jbsr.4060

**Published:** 2025-08-28

**Authors:** Caroline Chabot, Daniel Janssens

**Affiliations:** 1 Department of Radiology, Institut de Recherche Expérimentale et Clinique (IREC), Cliniques Universitaires Saint Luc, Université Catholique de Louvain, Brussels, Belgium; 2 Department of Radiology, Centre Hospitalier de Luxembourg, Luxembourg

**Keywords:** campanacci, fibula, imaging, osteofibrous dysplasia, tibia

## Abstract

*Teaching point:* Osteofibrous dysplasia (OFD) is a rare benign fibro‑osseous lesion primarily affecting the tibial cortex in children and adolescents; simultaneous involvement of the fibula is exceptional and may mimic more aggressive tumors such as adamantinoma.

## Clinical History

**Figure 1:**
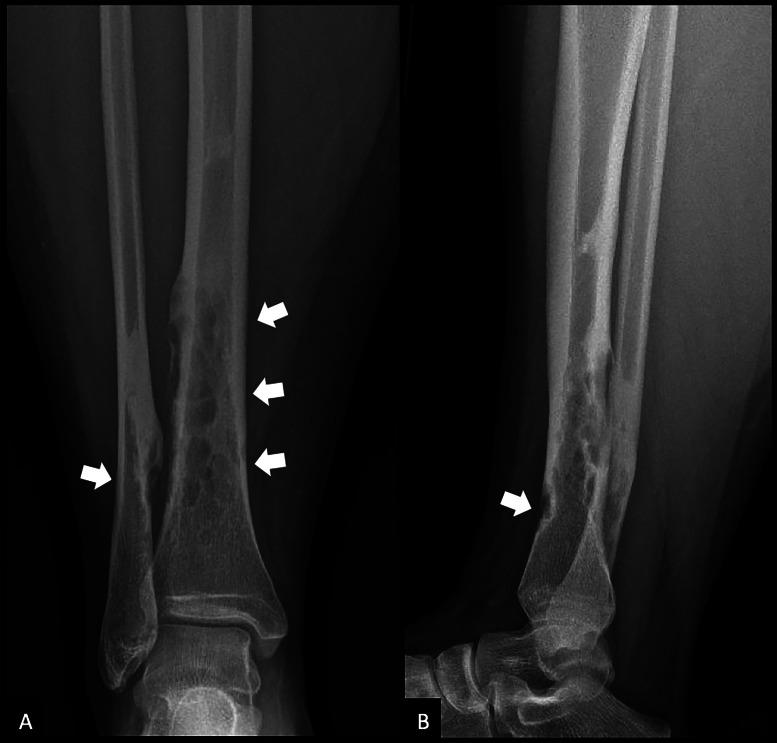
Conventional radiographs of the right leg, anteroposterior **(A)** and lateral **(B)** views. Cortical osteolytic lesions are visible in the distal third of the tibia and fibula (arrows). The lesions are well‑defined and associated with cortical thinning, without periosteal reaction or soft tissue involvement.

**Figure 2:**
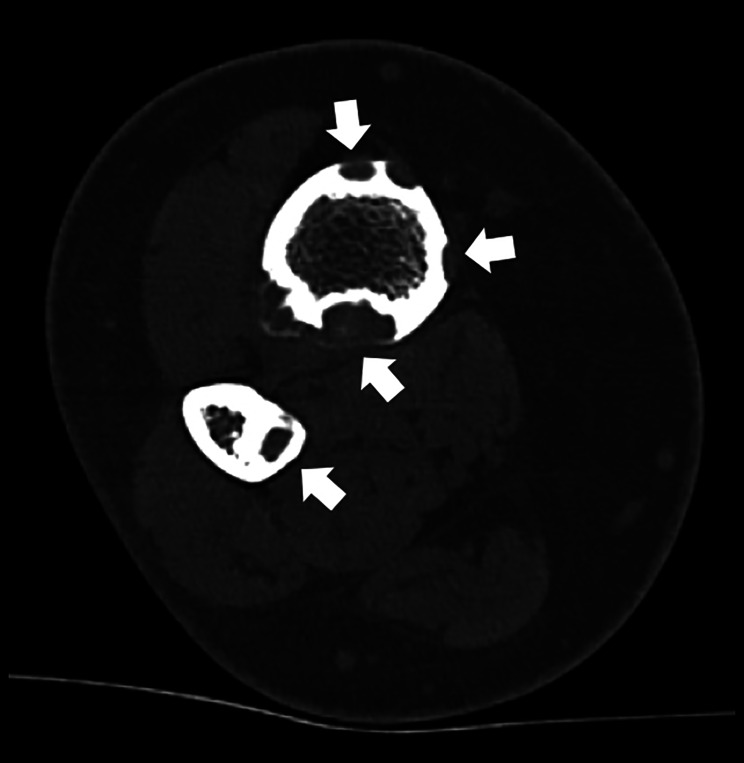
Axial CT image of the distal right leg showing well‑defined intracortical lucencies (arrows). Associated findings include cortical thinning and focal cortical defects, without evidence of periosteal reaction or extraosseous extension.

**Figure 3:**
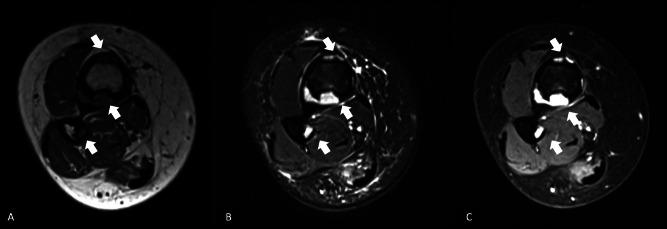
MRI of the distal right leg: T1‑weighted **(A)**, T2 DIXON WATER **(B)**, and post‑gadolinium contrast‑enhanced **(C)** sequences. Multiple intracortical lesions are centered in the cortex of the distal tibia, primarily involving the posterolateral and anterolateral aspects, with some extension into the anterior cortex (arrows). The lesions are hypointense on T1, hyperintense on T2 DIXON WATER, and show homogeneous enhancement after gadolinium administration. Additional small lesions are noted in the posteromedial cortex of the fibula.

A 20‑year‑old woman presented with chronic pain localized to the distal third of her right leg, evolving over several months. She had no significant medical history or trauma. Conventional radiographs revealed cortical osteolytic lesions in the distal tibia and fibula (Figure 1, arrows). The CT scan showed well‑circumscribed intracortical lucencies at the diaphyseal–metaphyseal junctions of both bones, with cortical thinning and focal defects. There was no evidence of a periosteal reaction or soft tissue mass (Figure 2, arrows). MRI demonstrated multiple focal, partly confluent lesions centered in the cortex of the distal tibia, predominantly involving the posterolateral and anterolateral aspects, with some extension into the anterior cortex. These lesions showed low signal intensity on T1‑weighted images, high signal on T2 DIXON WATER sequences, and homogeneous, diffuse enhancement after gadolinium administration. Millimetric lesions were also observed in the posteromedial cortex of the fibula (Figure 3, arrows). Overall, the imaging features suggested an intracortical lesion of subperiosteal origin that was largely contained within the bone contours and did not extend into the medullary cavity or soft tissues.

## Comments

First described by Campanacci in 1976, osteofibrous dysplasia (OFD) is a rare benign fibro‑osseous lesion that predominantly affects the mid‑diaphysis of the tibia in children. Accounting for around 0.2% of primary bone tumors, it typically presents before the age of 10, though cases in late adolescence or adulthood do occur. OFD is usually monostotic, involving a single bone. Simultaneous involvement of the tibia and fibula is uncommon, occurring in fewer than 12% of cases. In this case, the cortical lesions and the absence of soft tissue extension are consistent with multifocal monostotic disease [1].

Imaging is pivotal for diagnosis. Radiographs and CT scans usually reveal well‑defined intracortical osteolytic lesions with a sclerotic rim and no periosteal reaction. MRI shows lesions with an intermediate to low T1 signal, a high T2 signal, and homogeneous contrast enhancement. These features, in the absence of medullary or soft tissue involvement, suggest a benign fibro‑osseous lesion [1].

However, OFD imaging can mimic that of adamantinoma, a low‑grade malignant tumor predominantly affecting young adults and involving the tibial diaphysis. Adamantinoma often exhibits more aggressive features, including cortical destruction, medullary invasion, soft tissue extension, and skip lesions. Since imaging alone cannot always distinguish between OFD and differentiated adamantinoma, histopathology is often necessary. Adequate biopsy sampling is critical to prevent misdiagnosis. Immunohistochemistry may reveal scattered keratin‑positive epithelial cells in OFD, whereas adamantinoma is characterized by prominent epithelial nests [1].

In conclusion, this case illustrates a rare presentation of OFD involving both the tibia and fibula in a young adult. It is essential to recognize this benign entity and its imaging features in order to guide proper management and avoid overtreatment or misdiagnosis as malignancy.
